# Co‐occurrence of CDKN2A/B and IFN‐I homozygous deletions correlates with an immunosuppressive phenotype and poor prognosis in lung adenocarcinoma

**DOI:** 10.1002/1878-0261.13206

**Published:** 2022-03-15

**Authors:** Yuan Peng, Yonghong Chen, Mengmeng Song, Xiaoyue Zhang, Pansong Li, Xian Yu, Yusheng Huang, Ni Zhang, Liyan Ji, Lei Xia, Xuefeng Xia, Xin Yi, Benxu Tan, Zhenzhou Yang

**Affiliations:** ^1^ 12550 Department of Cancer Center Second Affiliated Hospital Chongqing Medical University Chongqing China; ^2^ Geneplus‐Beijing Institute Beijing China

**Keywords:** *CDKN2A/B*, homozygous deletion, tumor immune microenvironment, type I interferons

## Abstract

Homozygous deletion (HD) of *CDKN2A* and *CDKN2B* (*CDKN2A/B* ^HD^) is the most frequent copy‐number variation (CNV) in lung adenocarcinoma (LUAD). *CDKN2A/B* ^HD^ has been associated with poor outcomes in LUAD; however, the mechanisms of its prognostic effect remain unknown. We analyzed genome, transcriptome, and clinical data from 517 patients with LUAD from the Cancer Genome Atlas (TCGA) and from 788 primary LUAD tumor and matched control samples from the MSK‐IMPACT clinical cohort. *CDKN2A/B* ^HD^ was present in 19.1% of the TCGA‐LUAD cohort and in 5.7% of the MSK‐IMPACT cohort. *CDKN2A/B* ^HD^ patients had shorter disease‐free survival and overall survival compared with *CDKN2A/B* ^WT^ individuals in both cohorts. Differences in clinical features did not influence the outcomes in the CDKN2A/B^HD^ population. Mutation analyses showed that overall tumor mutational burden and mutations in classical drivers such as EGFR and RB1 were not associated with CDKN2A/B^HD^. In contrast, homozygous deletion of type I interferons (IFN‐I^HD^) frequently co‐occurred with CDKN2A/B^HD^. CDKN2A/B and IFN‐I are co‐located in the same p21.3 region of chromosome 9. The co‐occurrence of CDKN2A/B^HD^ and IFN‐I^HD^ was not related to whole‐genome doubling, chromosome instability, or aneuploidy. Patients with co‐occurring *CDKN2A/B* ^HD^ and *IFN‐I* ^HD^ had shorter disease‐free survival and overall survival compared with *CDKN2A/B* ^WT^ patients. *CDKN2A/B* ^HD^ *IFN‐I* ^HD^ had downregulated several key immune response pathways, suggesting that poor prognosis in *CDKN2A/B* ^HD^ LUAD could potentially be attributed to an immunosuppressive tumor microenvironment as a result of IFN‐I depletion.

## Graphical abstract

CDKN2A/B homozygous deletion (CDKN2A/B^HD^) was a common event and associated with poor outcomes in lung adenocarcinoma (LUAD). Homozygous deletion of type I interferons (IFN‐I^HD^) frequently co‐occurred with CDKN2A/B^HD^. CDKN2A/B^HD^IFN‐I^HD^ had shorter survival times than CDKN2A/B^WT^ and downregulated immune response pathways, suggesting that poor prognosis in CDKN2A/B^HD^ LUAD could potentially be attributed to an immunosuppressive tumor microenvironment owing to IFN‐I depletion.

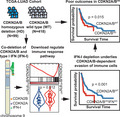

**Abbreviations**
CNV: copy‐number variation
DFS: disease‐free survival
GSEA: gene set enrichment analysis
HD: homozygous deletion
IFN‐I: type I interferons
LUAD: lung adenocarcinoma
NSCLC: non‐small cell lung carcinoma
OS: overall survival
TCGA: The Cancer Genome Atlas
TMB: tumor mutational burden
TSG: tumor suppressor gene

## 1 Introduction

As typical tumor suppressor genes (TSGs), the cyclin‐dependent kinase inhibitors *CDKN2A* and *CDKN2B* located on chromosome 9, band p21.3, are frequently mutated, deleted, or dysregulated in a variety of cancers [1, 2, 3, 4, 5]. Deficient function of TSGs leads to tumor proliferation and progression. Homozygous deletion (HD) and corresponding loss of function of *CDKN2A* and *CDKN2B* is associated with poor prognosis in diffuse malignant IDH‐mutant glioma [6], thymic carcinoma [7], pleural mesothelioma [8], urothelial bladder carcinoma [9], neuroblastoma [10], T cell acute lymphoblastic leukemia [11], and pancreatic cancer [12]. This suggests that *CDKN2A* and *CDKN2B* play an important role in certain cancer types.

Knowledge of the roles of *CDKN2A* and *CDKN2B* HD in lung adenocarcinoma (LUAD) is scarce. *CDKN2A* was mutated or homozygously deleted in 20 of 32 (63%) non‐small cell lung carcinoma (NSCLC) cell lines, and *CDKN2B* HD was also detected in the same lines [13]. Two‐hit inactivation of *CDKN2A*/*2B* was frequently found in *KRAS*‐mutant LUAD in The Cancer Genome Atlas (TCGA) database, and loss of *CDKN2A/B* fostered cellular proliferation, cancer cell differentiation, and metastatic behavior in genetically engineered mouse models of *KRAS*‐mutant lung tumorigenesis [14]. Recently, loss of *CDKN2A* function was found to be related to NSCLC clinical outcomes. *CDKN2A* HD was detected in 24.4% (31/127) of LUADs in a Chinese cohort, and the occurrence of *CDKN2A* HD in *EGFR*‐mutant LUADs was associated with poor response to EGFR tyrosine kinase inhibitors (TKIs) [15]. These findings support the significance of *CDKN2A* HD in the clinical management of LUAD; however, the mechanisms of *CDKN2A/B* HD and its effects on the tumor immune microenvironment have not been revealed.

In this study, we aimed to reveal the key genomic and immune‐related mechanisms of the prognostic effects of *CDKN2A* and *CDKN2B* HD on LUAD prognosis. We found that HD of type I interferon (*IFN*‐*I*) genes was the most frequent type of copy‐number variation (CNV) accompanying *CDKN2A* and *CDKN2B* HD in LUAD. A previous report suggested that homozygous co‐deletion of IFN‐I and CDKN2A is a potential biomarker for therapy in thoracic cancers [16]. There are 16 *IFN*‐*I* genes located on chromosome 9p21 that share a common receptor, induce immune response, and participate in cell antiviral and anti‐tumor defense. We found that co‐deletion of *IFN*‐*I* and *CDKN2A* or *CDKN2B* was associated with poor clinical outcomes and downregulated expression of genes related to inflammatory response, adaptive immune response, and JAK‐STAT signaling in the tumor microenvironment. These findings provide fundamental knowledge about LUAD with cyclin‐dependent kinase inhibitor dysfunction and indicate the necessity of tailored treatment for patients with this molecular subtype of lung cancer.

## 2 Materials and methods

### 2.1 Datasets from the TCGA and MSK‐IMPACT Cohorts

We used the TCGAbiolinks R package to download data of 517 TCGA‐LUAD primary tumor samples and matched nontumor samples, including somatic mutation and masked CNV segment data, RNA sequencing data, and clinical data [17]. We downloaded corresponding patient follow‐up data from the cBioportal database (http://www.cbioportal.org/). In addition, we downloaded data of 788 primary LUAD tumor samples and matched control samples from the MSK‐IMPACT clinical sequencing cohort using the cBioportal website.

### 2.2 Focal‐level and arm‐level identification

There were multiple primary tissue samples for some of the patients; however, to ensure consistency of the CDKN2A/B mutation status, we only used one primary tumor sample from each patient. If more than one sample was available for a given patient, we selected a single sample with HD of CDKN2A or CDKN2B (*CDKN2A/B^HD^*). In cases where a single patient had more than one sample with *CDKN2A/B^HD^*, we randomly selected one of these samples to use.

To identify genes with somatic CNV, we used GISTIC2 [18] with the following parameters: ‐ta 0.1, ‐armpeel 1, ‐brlen 0.7, ‐cap 1.5, ‐conf 0.99, ‐td 0.1, ‐genegistic 1, ‐gcm extreme, ‐js 4, ‐maxseg 2000, ‐qvt 0.25, ‐rx 0, ‐savegene 1, ‐broad 1, and all other parameters set to default values. The copy number for each gene was given in an *all_thresholded.by_genes.txt* file, with values of −2, −1, 0, 1, and 2 representing deep deletion (HD), shallow deletion, diploid, low‐level gain, and high‐level gain, respectively.

### 2.3 Mutation signature analysis

We extracted mutation signatures from the samples using the Sigminer R package. First, we used the *read_maf* method to load all the somatic mutations and tallied components in each sample. We then generated a sample‐by‐component matrix using the *sig_tally* method. Then, we used *sig_fit* to perform a signature decomposition of the mutation catalog and compute the absolute exposure of all COSMIC mutation signatures from the spectrum of each sample. This resulted in an absolute exposure matrix, in which the rows represented the samples and the columns represented the COSMIC signatures. We then used Fisher’s exact test to determine whether or not each signature was associated with *CDKN2A/B* CNV status.

### 2.4 Immunity analysis and gene set enrichment analysis

We performed immunity analysis using the GSVA [19] package and 25 previously reported immune‐related gene sets covering the innate and adaptive immune responses [20]. This produced an enrichment score for each immune‐related gene set in each sample. We used gene set enrichment analysis (GSEA) software to identify biological pathways that were differentially enriched (*P*‐value > 0.05 and absolute value of enrichment score > 1) between tumor molecular subtypes [21].

### 2.5 Statistical analysis

We performed Kaplan–Meier survival analyses implemented in the R package survival. We then used log‐rank tests to determine significant differences in survival curves. We reported median overall survival (OS) with 95% confidence intervals in relevant cases. We used Fisher’s exact tests to determine associations between genomic characteristics and clinical characteristics and to determine which mutations/CNV co‐occurred or were mutually exclusive with *CDKN2A/B* ^HD^. We used Mann–Whitney tests to compare differences between different tumor molecular subtypes. *P*‐values less than 0.05 were considered statistically significant.

## 3 Results

### 3.1 *CDKN2A/B* ^HD^ was highly recurrent and indicated poor prognosis in LUAD

We first investigated the prevalence of *CDKN2A* and *CDKN2B* HD in two independent LUAD cohorts. In the TCGA‐LUAD cohort, the frequencies of *CDKN2A* HD and *CDKN2B* HD were 19.0% (98/517) and 18.4% (95/517), respectively. Co‐deletion of both genes within the same patient was very common (*P* < 0.001, OR = 7572.3), so we decided to analyze both genes in combination. The patient characteristics of the TCGA‐LUAD cohort are described in Table S1. HD of CDKN2A or CDKN2B (*CDKN2A/B* ^HD^) was the most prevalent CNV event in the TCGA‐LUAD cohort, appearing in 19.1% (99/517) of the patients. *CDKN2A/B* ^HD^ was also one of the prevalent CNV events in the MSK‐IMPACT cohort, appearing in 5.7% (45/788) of the patients. These results confirmed that *CDKN2A/B* ^HD^ is a common genetic event in LUAD, which is consistent with previous reports [22].

**Fig. 1:**
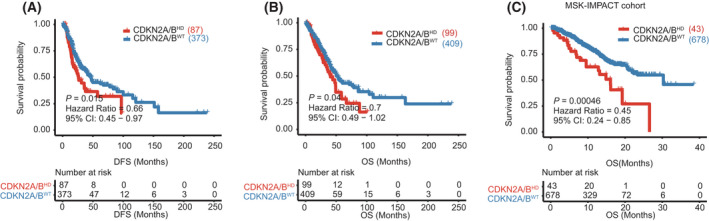
Relationship between *CDKN2A/B* homozygous deletion and survival in the TCGA‐LUAD cohort and the MSK‐IMPACT cohort. (A) Disease‐free survival in patients with *CDKN2A/B* homozygous deletion (*n* = 87) or wild‐type *CDKN2A/B* (*n* = 373) in the TCGA‐LUAD cohort. (B) Overall survival in patients with *CDKN2A/B* homozygous deletion (*n* = 99) or wild‐type *CDKN2A/B* (*n* = 409) in the TCGA‐LUAD cohort. (C) Overall survival in patients with *CDKN2A/B* homozygous deletion (*n* = 43) or wild‐type *CDKN2A/B* (*n* = 678) in the MSK‐IMPACT cohort. The log‐rank test was used to compare the survival times between two groups. A 95% confidence interval was used to indicate the precision of the estimated hazard ratio.

We next analyzed the potential influence of *CDKN2A/B* ^HD^ on LUAD outcomes, using disease‐fee survival (DFS) and OS as the primary endpoints. In the TCGA‐LUAD cohort, patients with *CDKN2A/B* ^HD^ tumors had significantly shorter DFS (*P* = 0.015, HR 0.66, 95% CI 0.45–0.97; Fig. 1A) and OS (*P* = 0.040, HR 0.70, 95% CI 0.49–1.02; Fig. 1B) than patients with wild‐type CDKN2A/B (*CDKN2A/B* ^WT^) tumors. *CDKN2A/B* ^HD^ was also associated with shortened OS in the MSK‐IMPACT cohort (*P* < 0.001, HR 0.45, 95% CI 0.24–0.85; Fig. 1C). The MSK‐IMPACT cohort did not provide DFS data. These results confirmed *CDKN2A/B* ^HD^ poor prognostic factor in LUAD.

### 3.2 *CDKN2A/B* ^HD^ tumors had disparate mutational features compared with CDKN2A/B^WT^ tumors

**Fig. 2:**
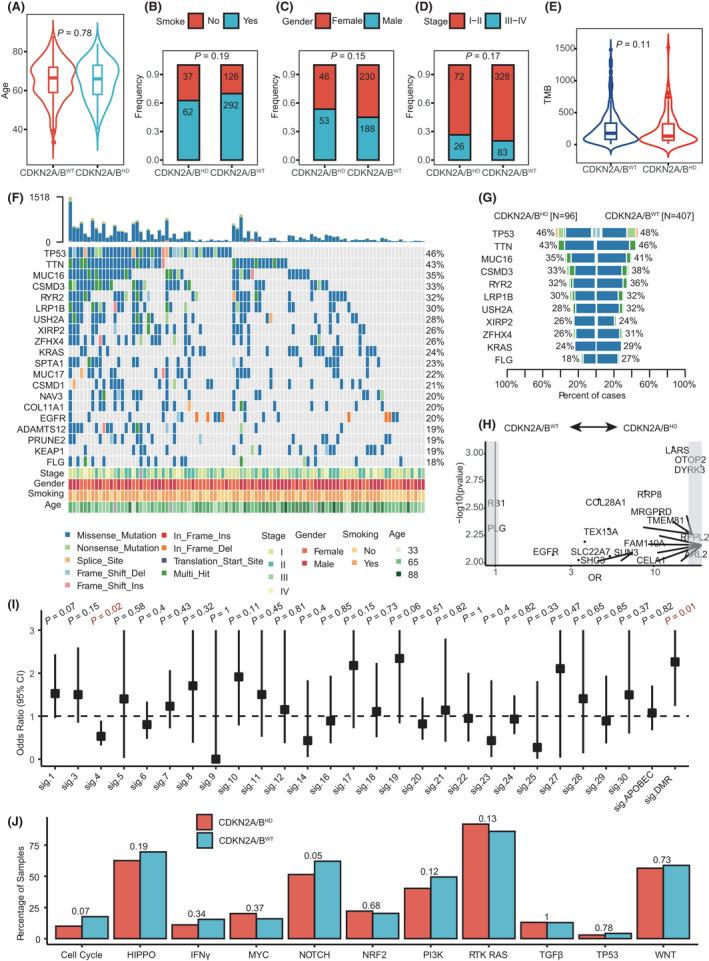
Comparison of clinical characteristics and genomic features between patients with *CDKN2A/B* homozygous deletion and patients with wild‐type *CDKN2A/B*. The associations between *CDKN2A/B* CNV status (*CDKN2A/B* homozygous deletion: *n* = 99, wild‐type *CDKN2A/B*: *n* = 418) and (A) age, (B) smoking history, (C) gender, and (D) tumor stage (*CDKN2A/B* homozygous deletion: *n* = 98, wild‐type *CDKN2A/B*: *n* = 411). (E) Comparison of mutational burden between tumors with *CDKN2A/B* homozygous deletion (*n* = 96) and tumors with wild‐type *CDKN2A/B* (*n* = 407). (F) The mutation landscape of tumors with *CDKN2A/B* homozygous deletion (*n* = 96). (G) Comparison of the frequencies of recurrently mutated genes between tumors with *CDKN2A/B* homozygous deletion (*n* = 96) and tumors with wild‐type *CDKN2A/B* (*n* = 407). (H) Mutations that co‐occurred or were mutually exclusive with *CDKN2A/B* homozygous deletion (*n* = 96, wild‐type *CDKN2A/B*: *n* = 407). (I) The distribution of mutational signatures in tumors with *CDKN2A/B* homozygous deletion (*n* = 96) and tumors with wild‐type *CDKN2A/B* (*n* = 407). The graph showed the estimates and 95% confidence intervals. (J) The distribution of mutant pathways in tumors with *CDKN2A/B* homozygous deletion (*n* = 96) and tumors with wild‐type *CDKN2A/B* (*n* = 407). (A, E) *P*‐values were calculated by Mann–Whitney test. The centerline of the boxplot represents the median, while the lower and upper limits of the box correspond to the 25th and 75th percentiles. Whiskers extend from the box limit to the minimum or maximum, not exceeding the 1.5 * quartile range. (B, C, D, H, I, J) *P*‐values were calculated by Fisher’s test.

To explore the prognostic mechanism of *CDKN2A/B* ^HD^ in LUAD, we first analyzed common clinical characteristics including age, gender, smoking history, and tumor stage. The results revealed no significant differences in clinical characteristics between patients with *CDKN2A/B* ^HD^ and patients with *CDKN2A/B* ^WT^ in the TCGA‐LUAD cohort (Figs. 2A‐D). Furthermore, although high tumor mutational burden (TMB) was associated with better prognosis in a previous study of patients with resected LUAD [23], there was no significant difference in TMB between *CDKN2A/B* ^HD^ tumors and *CDKN2A/B* ^WT^ tumors in the TCGA‐LUAD cohort (Fig. 2E).

We compared the genomic landscapes between *CDKN2A/B* ^HD^ tumors and *CDKN2A/B* ^WT^ tumors in the TCGA cohort to identify potentially prognostic genetic factors. The most frequently mutated genes, including *TP53* (46% vs. 48%), *TTN* (43% vs. 46%), *MUC16* (35% vs. 41%), and *CSMD3* (33% vs. 38%), had roughly equivalent mutation frequencies in both molecular subtypes of tumors (Fig. 2F). Also, the mutation frequencies of 11 genes that represented the union of the top 10 recurrently mutated genes in both tumor molecular subtypes were similar (Fig. 2G). We next used Fisher’s exact tests to comprehensively examine co‐occurring and mutually exclusive mutation events. The results showed that mutations in several genes either co‐occurred (e.g., *EGFR*) or were mutually exclusive (e.g., *RB1*) with CDKN2A/B^HD^ (Fig. 2H). Further analysis showed that co‐occurring or mutually exclusive mutations were not prognostic in the TCGA cohort (Fig. S1A,B), indicating that the prognostic effect of *CDKN2A/B* ^HD^ was not influenced by these mutations.

To identify the processes driving mutagenesis, we analyzed all the samples in the TCGA‐LUAD cohort to determine the proportion of mutations in each sample that were attributable to COSMIC mutational signatures (v2) on the basis of their flanking trinucleotide context. We then used Fisher’s exact test to test whether each COSMIC signature was associated with *CDKN2A/B* ^HD^. We found significant associations for three out of 30 COSMIC signatures: signature 4 (associated with tobacco use; OR = 0.53 [0.32–0.89], *P* = 0.02) and signatures 15 and 26 (associated with defective DNA mismatch repair; OR = 2.26 [1.24–4.05], *P* = 0.01; Fig. 2I).

We also compared mutations in 10 typical signaling pathways between *CDKN2A/B* ^HD^ tumors and *CDKN2A/B* ^WT^ tumors in the TCGA‐LUAD cohort. If a given pathway contained at least one mutated gene, then we considered the pathway to be mutated. We found that the NOTCH signaling pathway was more likely to mutated in *CDKN2A/B* ^WT^ tumors than in *CDKN2A/B* ^HD^ tumors (*P* = 0.05; Fig. 2J).

### 3.3 Variation causing loss of IFN‐I function was the most frequent co‐occurring CNV event with *CDKN2A/B* ^HD^

**Fig. 3:**
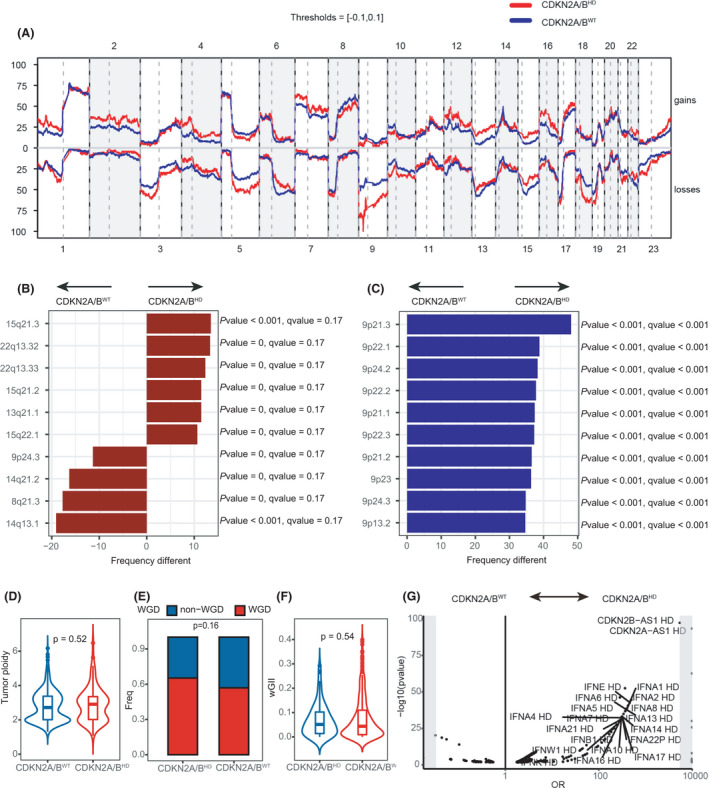
Genome‐wide somatic copy‐number variations in patients with wild‐type *CDKN2A/B* and patients with *CDKN2A/B* homozygous deletion. (A) The frequencies of genome‐wide somatic copy‐number gain (top) and loss (bottom) in tumors with *CDKN2A/B* homozygous deletion (*n* = 99, red line) and wild‐type *CDKN2A/B* (*n* = 418, blue line). Significantly different gain (B) or loss (C) frequencies of cytobands in tumors with *CDKN2A/B* homozygous deletion (*n* = 99) versus tumors with wild‐type *CDKN2A/B* (*n* = 418). Comparison of (D) tumor ploidy, (E) whole‐genome doubling, and (F) genome‐instability index between tumors with *CDKN2A/B* homozygous deletion (*n* = 99) and tumors with wild‐type *CDKN2A/B* (*n* = 418). (G) Copy‐number variation that was co‐occurring or mutually exclusive with *CDKN2A/B* homozygous deletion (*n* = 99). (B, C, E, G) *P*‐values were calculated by Fisher’s test. (D, F) The centerline of the boxplot represents the median, while the lower and upper limits of the box correspond to the 25th and 75th percentiles. Whiskers extend from the box limit to the minimum or maximum, not exceeding the 1.5 * quartile range. *P*‐values were calculated by Mann–Whitney test.

Next, we investigated the difference in CNV between *CDKN2A/B* ^HD^ tumors and *CDKN2A/B* ^WT^ tumors to identify potentially prognostic CNV events. The frequencies of copy‐number amplification and deletion in each chromosome region are shown in Fig. 3A. The results showed that the *CDKN2A/B* ^HD^ tumors had a high frequency of deletion in the chromosome 9p region. Differences in copy‐number amplifications between the *CDKN2A/B* ^HD^ tumors and the *CDKN2A/B* ^WT^ tumors mainly appeared on chromosomes 14 and 15 (Fig. 3B), whereas differences in copy‐number deletions appeared on chromosomes 5, 9, 12, 14, 18, 19, and 20. The most significant copy‐number deletions are shown in Fig. 3C. There were no significant differences in genome‐instability index, whole‐genome doubling, or tumor ploidy between the two groups (Fig. 3D,E,F). These results suggested that *CDKN2A/B* ^HD^ LUAD is not associated with broad chromosome‐level instability, which contributes to poor prognosis by accelerating the development of anticancer drug resistance [24].

**Fig. 4:**
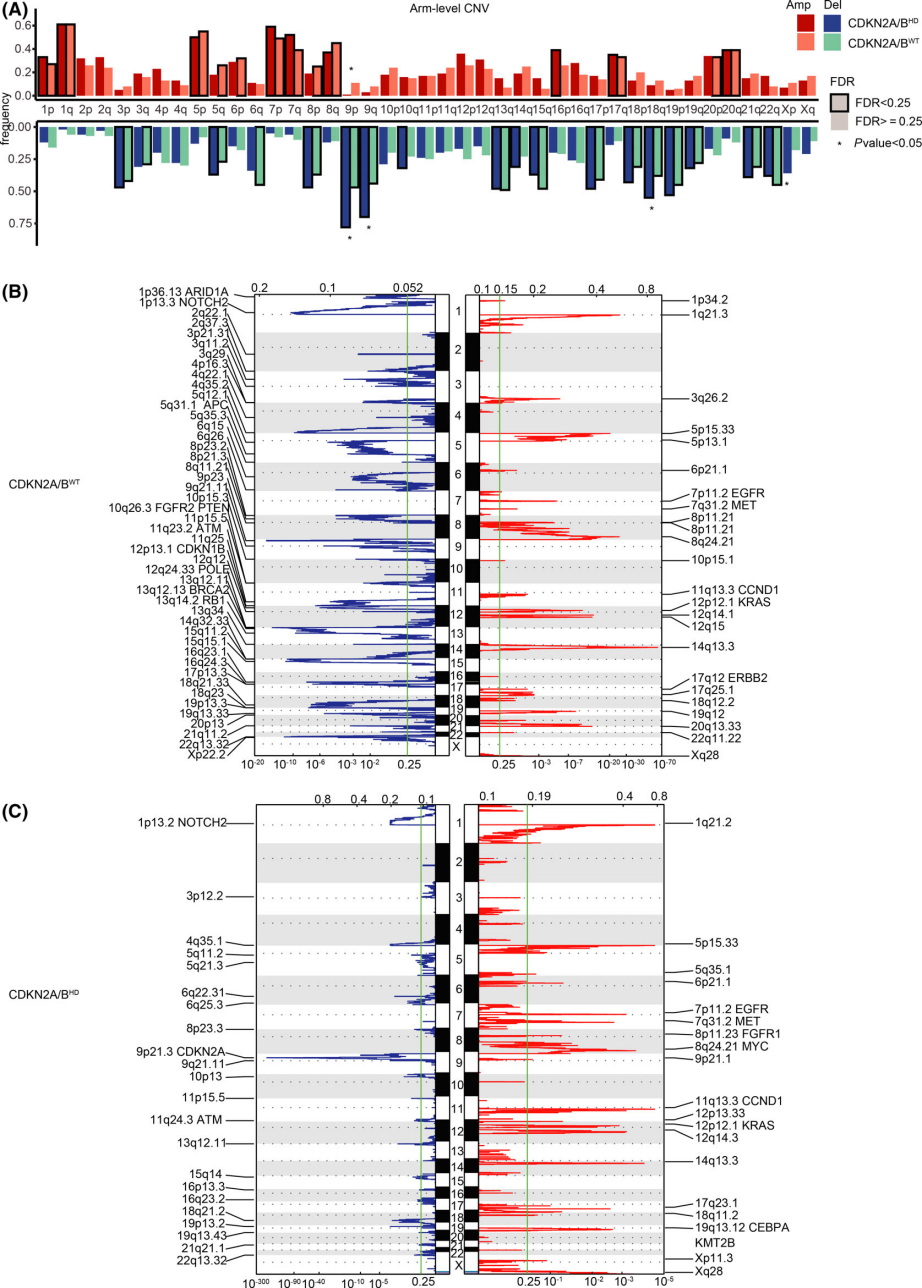
Significant arm‐level and focal somatic copy‐number variations in patients with wild‐type *CDKN2A/B* and patients with *CDKN2A/B* homozygous deletion. (A) Somatic CNV of arm‐level amplifications and deletions in *CDKN2A/B* wild‐type (*n* = 99) and *CDKN2A/B* homozygous deletion (*n* = 418). *P*‐values were calculated by Fisher’s test. (B) Somatic CNV of focal amplifications and deletions in *CDKN2A/B* wild‐type (*n* = 418) and (C) *CDKN2A/B* homozygous deletion (*n* = 99).

We further analyzed the focal and arm‐level copy‐number profiles of the *CDKN2A/B* ^HD^ and *CDKN2A/B* ^WT^ tumors using GISTIC2.0. The *CDKN2A/B* ^HD^ tumors showed a higher degree of arm‐level CNV than the *CDKN2A/B* ^WT^ tumors, and the difference was most pronounced in deletions including 9p, 9q, 18q, and Xp (Fig. 4A). We also identified 41 regions of significant focal‐level CNV in the *CDKN2A/B* ^HD^ tumors (*FDR* < 0.25; Fig. 4C), including 19 regions of recurrent amplification covering common drivers such as *EGFR*, *MET*, *FGFR1*, *MYC*, and *KRAS*, and 22 regions of recurrent deletion, which contained *NOTCH2*, *ATM*, and *CDKN2A*. The frequently mutated 9p21.3 region, where *CDKN2A* and *CDKN2B* are located, contains numerous *IFN*‐*I* genes, which were the sites of the most common homozygous deletions that co‐occurred with *CDKN2A/B* ^HD^ (Fig. 3G).

### 3.4 Poor outcomes in *CDKN2A/B* ^HD^ LUAD were associated with *IFN*‐*I* ^HD^ genetic events

**Fig. 5:**
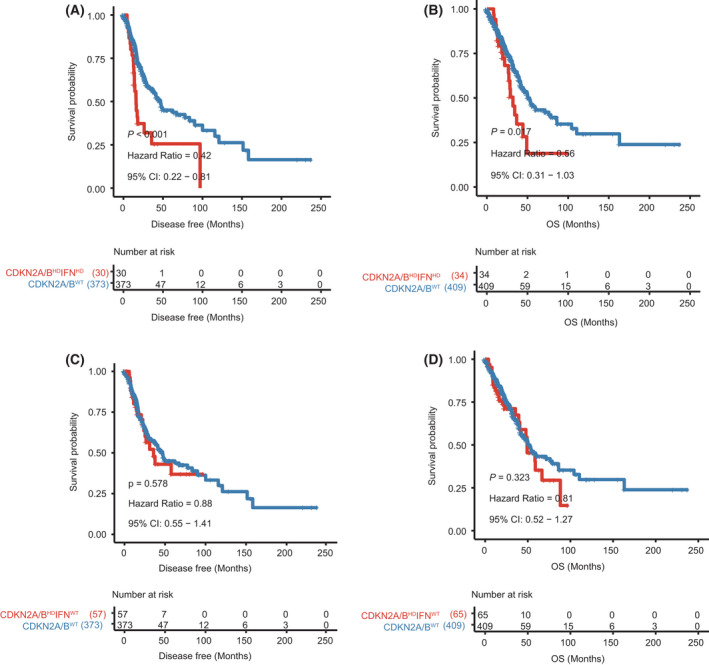
Kaplan–Meier curves comparison of disease‐free survival and overall survival between tumors with wild‐type *CDKN2A/B* and tumors with *CDKN2A/B* homozygous deletion and wild‐type IFN‐I/IFN‐I homozygous deletion. Differences in (A) disease‐free survival and (B) overall survival between patients with homozygous deletion of both *CDKN2A/B* and IFN‐I (A: *n* = 30, B: *n* = 34) and patients with wild‐type *CDKN2A/B* (A: *n* = 373, B: *n* = 409). Differences in (C) disease‐free survival and (D) overall survival between patients with *CDKN2A/B* homozygous deletion and wild‐type IFN‐I (C: *n* = 57, D: *n* = 65) and patients with wild‐type *CDKN2A/B* (C: *n* = 373, D: *n* = 409). (A, B, C, D) The log‐rank test was used to compare the survival times between two groups. A 95% confidence interval was used to indicate the precision of the estimated hazard ratio.

IFN‐I is a proinflammatory cytokine induced by viruses and other environmental stressors. It is also an important driver of anti‐tumor immunity, potentially enhancing the ability of immune cells to clear tumor cells [25]. Therefore, we asked whether *IFN*‐*I* variation was associated with outcomes in *CDKN2A/B* ^HD^ LUAD. We compared survival among patients with *CDKN2A/B* ^WT^ tumors and patients with *CDKN2A/B* ^HD^ tumors with or without accompanying homozygous deletion in all *IFN*‐*I* genes (*CDKN2A/B* ^HD^ *IFN*‐*I* ^HD^ and *CDKN2A/B* ^HD^ *IFN*‐*I* ^WT^, respectively). We found that the patients with *CDKN2A/B* ^HD^ *IFN*‐*I* ^HD^ tumors had shorter DFS (*P* < 0.001, HR 0.42, 95% CI 0.22–0.81) and OS (*P* = 0.02, HR 0.56, 95% CI 0.31–1.03) than the patients with *CDKN2A/B* ^WT^ tumors (Fig. 5A), whereas there was no difference between the patients with *CDKN2A/B* ^HD^ *IFN*‐*I* ^WT^ tumors and the patients with *CDKN2A/B* ^WT^ tumors in DFS (*P* = 0.58, HR 0.88, 95% CI 0.55–1.41; Fig. 5C) or OS (*P* = 0.32, HR 0.81, 95% CI 0.52–1.27; Fig. 5D). These results indicated that concomitant functional deletions of *IFN*‐*I* genes contribute to the prognosis of *CDKN2A/B* ^HD^ LUAD.

### 3.5 Suppression of the tumor immune microenvironment contributed to poor prognosis in *CDKN2A/B* ^HD^ *IFN*‐*I* ^HD^ LUAD

**Fig. 6:**
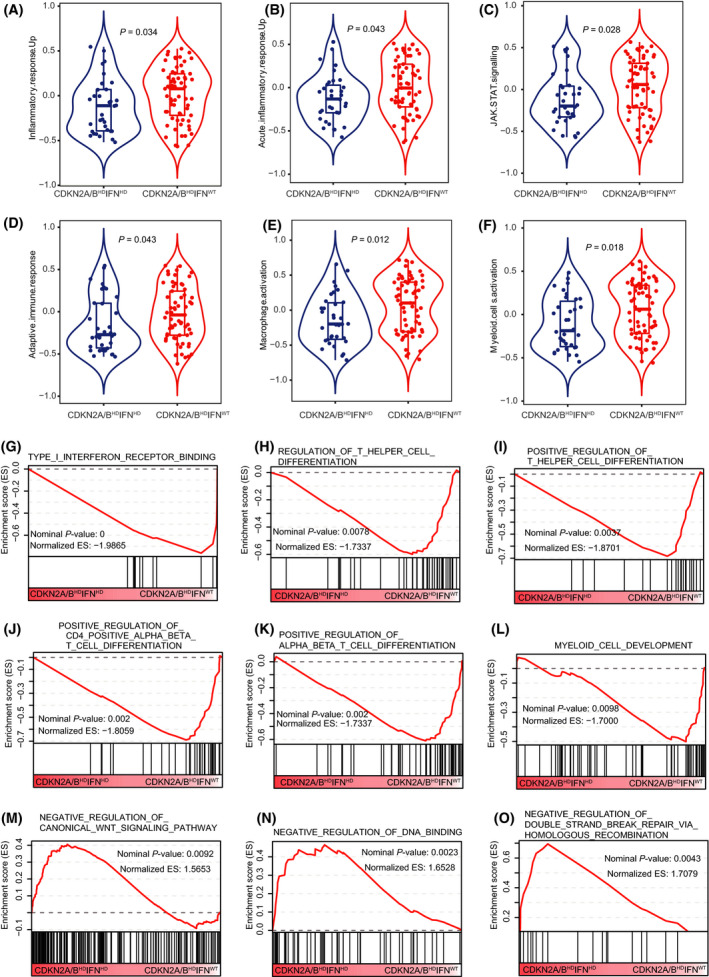
Comparison of immune‐related gene sets and pathway enrichment analysis between two different IFN‐I CNV statuses in patients with *CDKN2A/B* homozygous deletion. (A–F) Comparison of immune‐related gene sets between tumors with homozygous deletion of both *CDKN2A/B* and IFN‐I (*n* = 34) and tumors with *CDKN2A/B* homozygous deletion and wild‐type IFN‐I (*n* = 65). The centerline of the boxplot represents the median, while the lower and upper limits of the box correspond to the 25th and 75th percentiles. Whiskers extend from the box limit to the minimum or maximum, not exceeding the 1.5 * quartile range. *P*‐values were calculated by Mann–Whitney test. (G–L) Pathways with significant enrichment in tumors with homozygous deletion of both *CDKN2A/B* and IFN‐I. (M‐O) Pathways with significant enrichment in tumors with *CDKN2A/B* homozygous deletion and wild‐type IFN‐I.

It was reported that *IFN*‐*I* ^HD^ in human cancer was associated with immunotherapy resistance [26]. To further explore how the co‐deletion of *IFN*‐*I* influences outcomes in *CDKN2A/B* ^HD^ LUAD, we examined the tumor immune microenvironment by performing an immunity estimation of 25 gene sets associated with innate and adaptive immunity. A detailed gene list of the 25 gene sets is shown in Table S2. Comparison of the enrichment scores for the 25 gene sets between *CDKN2A/B* ^HD^ *IFN*‐*I* ^HD^ tumors and *CDKN2A/B* ^HD^ *IFN*‐*I* ^WT^ tumors showed that six immune‐related gene sets were relatively downregulated in the *CDKN2A/B* ^HD^ *IFN*‐*I* ^HD^ tumors, including signatures related to inflammatory response, acute inflammatory response, JAK‐STAT signaling, adaptive immune response, macrophage activation, and myeloid cell activation (Fig. 6A‐F). The results for the other 19 gene sets are shown in Fig. S2.

**Fig. 7:**
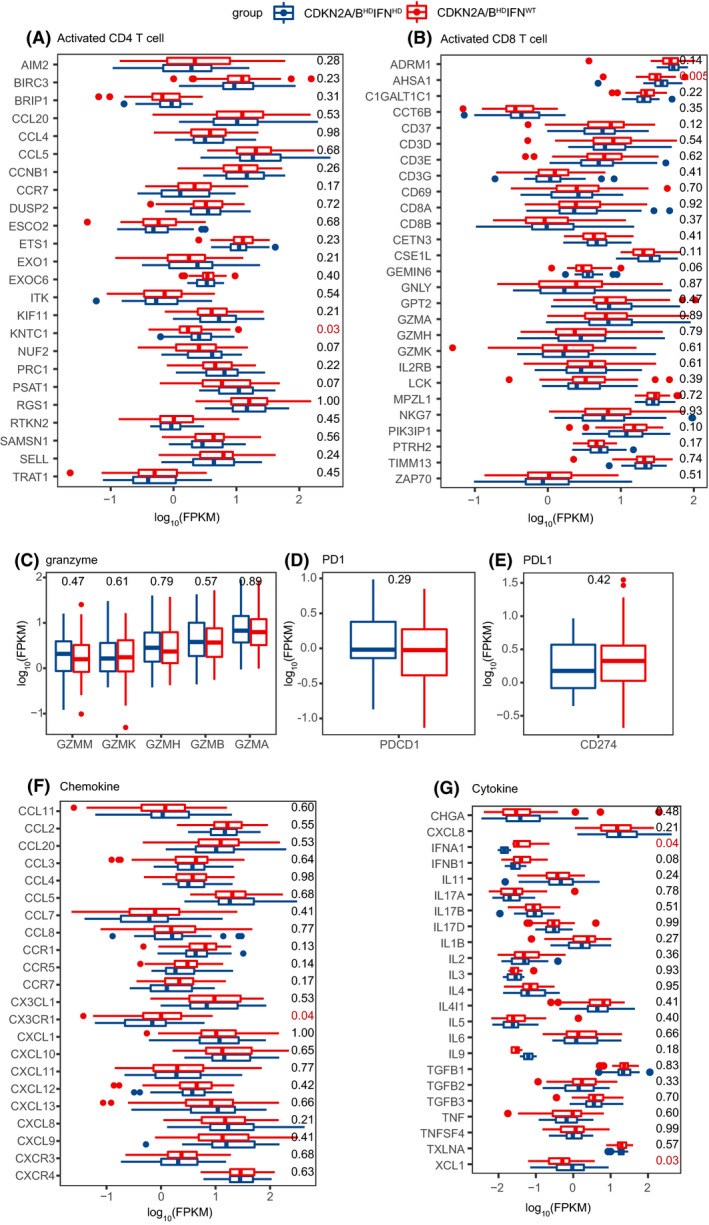
Boxplots for gene expression of immune‐related biomarkers. (A) activated CD4+ T cell, (B) activated CD8+ T cell, (C) granzymes, (D) PD1, (E) PD‐L1, (F) chemokines, and (G) cytokines between tumors with homozygous deletion of both *CDKN2A/B* and IFN‐I (*n* = 34) and tumors with *CDKN2A/B* homozygous deletion and wild‐type IFN‐I (*n* = 65). The centerline of the boxplot represents the median, while the lower and upper limits of the box correspond to the 25th and 75th percentiles. Whiskers extend from the box limit to the minimum or maximum, not exceeding the 1.5 * quartile range. *P*‐values were calculated by Mann–Whitney test.

A pathway enrichment analysis based on the GSEA results showed that negative regulation of the canonical WNT signaling pathway, negative regulation of DNA binding, and negative regulation of double‐strand break repair via homologous recombination were enriched in the *CDKN2A/B* ^HD^ *IFN*‐*I* ^HD^ tumors compared with the *CDKN2A/B* ^HD^ *IFN*‐*I* ^WT^ tumors. In the *CDKN2A/B* ^HD^ *IFN*‐*I* ^WT^ tumors, IFN‐I receptor binding, T helper cell differentiation, Tαβ cell differentiation, CD4αβ T cell differentiation, and myeloid cell development were upregulated (Fig. 6G,O). *IFN*‐*I* can activate the STAT3/4‐granzyme B pathway in tumor‐infiltrating CD8+ T cells, inhibit tumor growth [27, 28], and directly maintain the clonal expansion of CD4 T cells to fight virus infection [29]. We compared several key marker genes in activated CD4+ T cells, activated CD8+ T cells, and granzymes between *CDKN2A/B* ^HD^ *IFN*‐*I* ^WT^ tumors and *CDKN2A/B* ^HD^ *IFN*‐*I* ^HD^ tumors. *KNTC1* (marker for activated CD4+ T cell) and *AHSA1* (marker for activated CD8+ T cell) had significantly higher expression levels in *CDKN2A/B^HD^IFN*‐*I^HD^* tumors, whereas there were no significant differences in granzymes genes (Fig. 7A,B,C). *KNTC1* knockdown was previously shown to suppress cell proliferation and viability in various cancers [30, 31, 32]. *AHSA1* is a therapeutic target for the treatment of multiple myeloma [33]. IFN‐I signaling pathways in tumor cells are associated with the efficacy of immune checkpoint (such as PD1 and PD‐L1) inhibitor immunotherapy [34, 35]. However, our results showed that PD1/PD‐L1 expression was not significantly different between *CDKN2A/B* ^HD^ *IFN*‐*I* ^WT^ tumors and *CDKN2A/B* ^HD^ *IFN*‐*I* ^HD^ tumors (Fig. 7D,E). Innate immune cells respond to type I IFNs by enhancing antigen presentation and production of immune response mediators such as cytokines and chemokines [25, 36]. Expression of the chemokine receptor *CX3CR1*, which has a major role in proinflammatory and anti‐inflammatory responses [37], was lower in *CDKN2A/B* ^HD^ *IFN*‐*I* ^HD^ tumors than in *CDKN2A/B* ^HD^ *IFN*‐*I* ^WT^ tumors (Fig. 7F). We observed similar results for *IFNA1*. Conversely, expression of *XCL1*, which when produced by tumor cells may induce PD1/PD‐L1 interaction and dysfunction of CD8+ T cells in the tumor microenvironment [38], was higher in *CDKN2A/B* ^HD^ *IFN*‐*I* ^HD^ tumors than in *CDKN2A/B* ^HD^ *IFN*‐*I* ^WT^ tumors (Fig. 7G).

These results indicated that *IFN*‐*I* co‐deletion contributed to poor outcomes in *CDKN2A/B* ^HD^ LUAD by altering the tumor immune microenvironment.

## 4 Discussion

Recent studies suggested that *CDKN2A* ^HD^ is one of the most frequent genetic alterations in many human cancers, including LUAD [39]. Loss of *CDKN2A* has been associated with poor clinical prognosis and tumor progression in lung cancer [40]. However, the mechanism by which *CDKN2A/B* ^HD^ leads to poor prognosis has not yet been revealed. We analyzed the genomic events, tumor microenvironment characteristics, and clinical outcomes associated with *CDKN2A/B* ^HD^ LUAD and identified a mechanism involving IFN‐I that leads to poor prognosis.

We confirmed that *CDKN2A/B* ^HD^ LUAD was associated with worse outcomes than *CDKN2A/B* ^WT^ LUAD. Patients in the TCGA and MSK‐IMPACT cohorts with *CDKN2A/B* ^HD^ LUAD had shorter OS than with patients with *CDKN2A/B* ^WT^ LUAD. These results were consistent with those of previous lung cancer studies [15, 40]. Indeed, prognostic effects of *CDKN2A/B* ^HD^ have been observed in a series of cancers [6, 7, 8, 9, 10, 11, 12]. A pan‐cancer study of chromosome arm‐level CNV found that deletions on the 9p arm, which contains the *CDKN2A/B* genes, were among the most substantial arm‐level events in 33 cancer types [41]. Further survival analysis based on a Cox proportional hazard model revealed that *CDKN2A/B* copy‐number loss was one of the most significant prognosis‐related factors in low‐grade glioma. Therefore, we hypothesized that *CDKN2A/B* should be considered in the management of clinical lung cancer.

A previous study indicated that *CDKN2A/B* ^HD^ influenced the EGFR‐TKI response [15]. Although our comprehensive screening of the genomic landscape identified mutation events that either co‐occurred or were mutually exclusive with *CDKN2A/B* ^HD^ in LUAD, none of these events had any prognostic value in the TCGA cohort. Therefore, additional cohort data are needed to study the interaction between *CDKN2A/B* ^HD^ and *EGFR* in different treatment backgrounds.

Analysis of gene copy numbers revealed a potential prognostic mechanism for *CDKN2A/B* ^HD^ in LUAD. We found no prognostic influence of chromosome instability, whole‐genome doubling, or tumor ploidy, all of which were previously associated with accelerating resistance to anticancer chemotherapy, targeted therapy, and immunotherapy [24, 42, 43]. However, functional deletions of segmentally adjacent *IFN*‐*I* genes frequently co‐occurred with *CDKN2A/B* ^HD^, affecting 34.3% of the *CDKN2A/B* ^HD^ LUADs in TCGA cohort. Patients with *CDKN2A/B* ^HD^ *IFN*‐*I* ^HD^ tumors, but not those with *CDKN2A/B* ^HD^ *IFN*‐*I* ^WT^ tumors, had worse outcomes than patients with *CDKN2A/B* ^WT^ tumors, indicating a key role of IFN‐I dysfunction in determining the prognosis of *CDKN2A/B* ^HD^ LUAD.

Recent studies showed that IFN‐I is a crucial effector cytokine involved in antiviral immunity and mediates antineoplastic effects against several malignancies, which were attributed to its immunostimulatory functions [25]. Our analysis showed that functional damage to IFN‐I negatively regulated several immune responses, including T lymphocyte differentiation, IFN‐I receptor binding, inflammatory response, adaptive immune response, and JAK‐STAT signaling. IFN‐I and IFN‐I receptor heterodimer function as activators of JAK‐STAT signaling, which results in the recruitment of immune‐related signal transducers [44]. These results indicated that loss of IFN‐I function leads to a series of immune response signaling disorders. Furthermore, the clinical activity of a wide range of chemotherapeutic, radiotherapeutic, and immunotherapeutic interventions relies on the induction of IFN‐I signaling in malignant cells, tumor‐infiltrating myeloid cells, or lymphoid organs [25]. Accordingly, our results suggest that reduced myeloid cell activation might be related to IFN‐I deletion. In addition, *CDKN2A/B* ^HD^ *IFN* ^HD^ tumors were associated with canonical WNT signaling pathway negative regulation. Disorganization of canonical WNT signaling should be considered as a prognostic mechanism in cancer, as persistent WNT pathway activation was found to endow cancer cells with self‐renewing properties and was linked to therapy resistance [45].

We found that the negative prognostic effect of CDKN2A/B^HD^ in LUAD was dependent on loss of IFN‐I function. It has been reported that oncogenes such as *MYC* and *KRAS* can regulate immune response by suppressing IFN‐I pathways in various cancers. For example, the combined actions of endogenously expressed mutant KRAS and modestly deregulated *MYC* expression led to NK cell‐mediated immune escape through inhibition of IFN‐I pathways in pancreatic ductal adenocarcinoma and lung cancer [46, 47]. Overexpression of *MYC* suppresses the recruitment and activation of immune cells by inhibiting the induction of interferon signaling in triple‐negative breast cancer [48]. These studies further confirmed that repression of the type I Interferon pathways underlies oncogenes or tumor suppressor genes‐dependent evasion of immune cells in lung cancer.

## 5 Conclusions

We showed that *CDKN2A/B* ^HD^ is associated with poor prognosis in LUAD because of frequent co‐occurrence of IFN‐I functional loss, which leads to a suppressed tumor immune microenvironment.

## Conflict of interest

The authors declare no conflict of interest.

### Peer review

The peer review history for this article is available at https://publons.com/publon/10.1002/1878‐0261.13206.

## Author contributions

The conception and design of the study were undertaken by ZZY, BXT, XFX, and XY. Data were downloaded and processed by XYZ, XY, YSH, NZ, and LX. Data analysis and interpretation were performed by MMS and LYJ. Figures were prepared by YP and YHC. MMS, PSL, and YP wrote the manuscript. All authors approved the manuscript.

## Supporting information


**Fig S1**. Survival of patients with *CDKN2A/B* homozygous deletion and co‐occurring mutation in other genes. The log‐rank test was used to compare the survival times between two groups.

**Fig S2**. Comparison of immune‐related gene sets with no significant difference in enrichment score between two different IFN‐I CNV statuses in patients with *CDKN2A/B* homozygous deletion.

**Table S1**. Patient characteristics of the TCGA‐LUAD cohort.

**Table S2**. Detailed gene list of the 25 immune‐related gene sets.

## Data Availability

The data that support the findings of this study are available in TCGA at https://www.cancer.gov/about‐nci/organization/ccg/research/structural‐genomics/tcga.

## References

[1] Jafri M , Wake NC , Ascher DB , Pires DE , Gentle D , Morris MR , et al. Germline Mutations in the CDKN2B tumor suppressor gene predispose to renal cell carcinoma. Cancer Discov. 2015;5:723–9. 10.1158/2159-8290.Cd-14-1096. 25873077

[2] Liggett WH , Sidransky D . Role of the p16 tumor suppressor gene in cancer. J Clin Oncol. 1998;16:1197–206. 9508208 10.1200/JCO.1998.16.3.1197

[3] Yu W , Gius D , Onyango P , Muldoon‐Jacobs K , Karp J , Feinberg AP , et al. Epigenetic silencing of tumour suppressor gene p15 by its antisense RNA. Nature. 2008;451:202–6. 10.1038/nature06468. 18185590 PMC2743558

[4] Karayan L , Riou JF , Séité P , Migeon J , Cantereau A , Larsen CJ . Human ARF protein interacts with topoisomerase I and stimulates its activity. Oncogene. 2001;20:836–48. 10.1038/sj.onc.1204170. 11314011

[5] Zhao R , Choi BY , Lee MH , Bode AM , Dong Z . Implications of genetic and epigenetic alterations of CDKN2A (p16(INK4a)) in cancer. EBioMedicine. 2016;8:30–9. 10.1016/j.ebiom.2016.04.017. 27428416 PMC4919535

[6] Appay R , Dehais C , Maurage CA , Alentorn A , Carpentier C , Colin C , et al. CDKN2A homozygous deletion is a strong adverse prognosis factor in diffuse malignant IDH‐mutant gliomas. Neuro Oncol. 2019;21:1519–28. 10.1093/neuonc/noz124. 31832685 PMC7145561

[7] Aesif SW , Aubry MC , Yi ES , Kloft‐Nelson SM , Jenkins SM , Spears GM , et al. Loss of p16(INK4A) Expression and Homozygous CDKN2A deletion are associated with worse outcome and younger age in thymic carcinomas. J Thorac Oncol. 2017;12:860–71. 10.1016/j.jtho.2017.01.028. 28179162

[8] Illei PB , Rusch VW , Zakowski MF , Ladanyi M . Homozygous deletion of CDKN2A and codeletion of the methylthioadenosine phosphorylase gene in the majority of pleural mesotheliomas. Clin Cancer Res. 2003;9:2108–13. 12796375

[9] Rebouissou S , Hérault A , Letouzé E , Neuzillet Y , Laplanche A , Ofualuka K , et al. CDKN2A homozygous deletion is associated with muscle invasion in FGFR3‐mutated urothelial bladder carcinoma. J Pathol. 2012;227:315–24. 10.1002/path.4017. 22422578

[10] Thompson PM , Maris JM , Hogarty MD , Seeger RC , Reynolds CP , Brodeur GM , et al. Homozygous deletion of CDKN2A (p16INK4a/p14ARF) but not within 1p36 or at other tumor suppressor loci in neuroblastoma. Cancer Res. 2001;61:679–86. 11212268

[11] Genescà E , Lazarenkov A , Morgades M , Berbis G , Ruíz‐Xivillé N , Gómez‐Marzo P , et al. Frequency and clinical impact of CDKN2A/ARF/CDKN2B gene deletions as assessed by in‐depth genetic analyses in adult T cell acute lymphoblastic leukemia. J Hematol Oncol. 2018;11:96. 10.1186/s13045-018-0639-8. 30041662 PMC6057006

[12] Tu Q , Hao J , Zhou X , Yan L , Dai H , Sun B , et al. CDKN2B deletion is essential for pancreatic cancer development instead of unmeaningful co‐deletion due to juxtaposition to CDKN2A. Oncogene. 2018;37:128–38. 10.1038/onc.2017.316. 28892048 PMC5759028

[13] Hamada K , Kohno T , Kawanishi M , Ohwada S , Yokota J . Association of CDKN2A(p16)/CDKN2B(p15) alterations and homozygous chromosome arm 9p deletions in human lung carcinoma. Genes Chromosomes Cancer. 1998;22:232–40. 9624535 10.1002/(sici)1098-2264(199807)22:3<232::aid-gcc9>3.0.co;2-x

[14] Schuster K , Venkateswaran N , Rabellino A , Girard L , Peña‐Llopis S , Scaglioni PP . Nullifying the CDKN2AB locus promotes mutant K‐ras lung tumorigenesis. Mol Cancer Res. 2014;12:912–23. 10.1158/1541-7786.MCR-13-0620-T. 24618618 PMC4058359

[15] Jiang J , Gu Y , Liu J , Wu R , Fu L , Zhao J , et al. Coexistence of p16/CDKN2A homozygous deletions and activating EGFR mutations in lung adenocarcinoma patients signifies a poor response to EGFR‐TKIs. Lung Cancer. 2016;102:101–7. 10.1016/j.lungcan.2016.10.015. 27987577

[16] Grard M , Chatelain C , Delaunay T , Pons‐Tostivint E , Bennouna J , Fonteneau JF . Homozygous Co‐deletion of type I Interferons and CDKN2A genes in thoracic cancers: potential consequences for therapy. Front Oncol. 2021;11:695770. 10.3389/fonc.2021.695770. 34249754 PMC8266377

[17] Colaprico A , Silva TC , Olsen C , Garofano L , Cava C , Garolini D , et al. TCGAbiolinks: an R/Bioconductor package for integrative analysis of TCGA data. Nucleic Acids Res. 2016;44:e71. 10.1093/nar/gkv1507. 26704973 PMC4856967

[18] Mermel CH , Schumacher SE , Hill B , Meyerson ML , Beroukhim R , Getz G . GISTIC2.0 facilitates sensitive and confident localization of the targets of focal somatic copy‐number alteration in human cancers. Genome Biol. 2011;12:R41. 10.1186/gb-2011-12-4-r41. 21527027 PMC3218867

[19] Hänzelmann S , Castelo R , Guinney J . GSVA: gene set variation analysis for microarray and RNA‐seq data. BMC Bioinformatics. 2013;14:7. 10.1186/1471-2105-14-7. 23323831 PMC3618321

[20] García‐Mulero S , Alonso MH , Pardo J , Santos C , Sanjuan X , Salazar R , et al. Lung metastases share common immune features regardless of primary tumor origin. J Immunother Cancer. 2020;8:e000491. 10.1136/jitc-2019-000491. 32591432 PMC7319789

[21] Subramanian A , Tamayo P , Mootha VK , Mukherjee S , Ebert BL , Gillette MA , et al. Gene set enrichment analysis: a knowledge‐based approach for interpreting genome‐wide expression profiles. Proc Natl Acad Sci USA. 2005;102:15545–50. 10.1073/pnas.0506580102. 16199517 PMC1239896

[22] Shih DJH , Nayyar N , Bihun I , Dagogo‐Jack I , Gill CM , Aquilanti E , et al. Genomic characterization of human brain metastases identifies drivers of metastatic lung adenocarcinoma. Nat Genet. 2020;52:371–7. 10.1038/s41588-020-0592-7. 32203465 PMC7136154

[23] Devarakonda S , Rotolo F , Tsao MS , Lanc I , Brambilla E , Masood A , et al. Tumor mutation burden as a biomarker in resected non‐small‐cell lung cancer. J Clin Oncol. 2018;36:2995–3006. 10.1200/jco.2018.78.1963. 30106638 PMC6804865

[24] Sansregret L , Vanhaesebroeck B , Swanton C . Determinants and clinical implications of chromosomal instability in cancer. Nat Rev Clin Oncol. 2018;15:139–50. 10.1038/nrclinonc.2017.198. 29297505

[25] Zitvogel L , Galluzzi L , Kepp O , Smyth MJ , Kroemer G . Type I interferons in anticancer immunity. Nat Rev Immunol. 2015;15:405–14. 10.1038/nri3845. 26027717

[26] Ye Z , Dong H , Li Y , Ma T , Huang H , Leong HS , et al. Prevalent homozygous deletions of Type I interferon and defensin genes in human cancers associate with immunotherapy resistance. Clin Cancer Res. 2018;24:3299–308. 10.1158/1078-0432.Ccr-17-3008. 29618619 PMC6050078

[27] Lu C , Klement JD , Ibrahim ML , Xiao W , Redd PS , Nayak‐Kapoor A , et al. Type I interferon suppresses tumor growth through activating the STAT3‐granzyme B pathway in tumor‐infiltrating cytotoxic T lymphocytes. J Immunother Cancer. 2019;7:157. 10.1186/s40425-019-0635-8. 31228946 PMC6589175

[28] Newby BN , Brusko TM , Zou B , Atkinson MA , Clare‐Salzler M , Mathews CE . Type 1 interferons potentiate human CD8+ T‐cell cytotoxicity through a STAT4‐ and granzyme B‐dependent pathway. Diabetes. 2017;66:3061–71. 10.2337/db17-0106. 28877912 PMC5697952

[29] McNab F , Mayer‐Barber K , Sher A , Wack A , O'Garra A . Type I interferons in infectious disease. Nat Rev Immunol. 2015;15:87–103. 10.1038/nri3787. 25614319 PMC7162685

[30] Huang H , Fan X , Qiao Y , Yang M , Ji Z . Knockdown of KNTC1 Inhibits the proliferation, migration and tumorigenesis of human bladder cancer cells and induces apoptosis. Crit Rev Eukaryot Gene Expr. 2021;31:49–60. 10.1615/CritRevEukaryotGeneExpr.2021037301. 33639055

[31] Liu CT , Min L , Wang YJ , Li P , Wu YD , Zhang ST . shRNAmediated knockdown of KNTC1 suppresses cell viability and induces apoptosis in esophageal squamous cell carcinoma. Int J Oncol. 2019;54:1053–60. 10.3892/ijo.2019.4672. 30628654

[32] Zhengxiang Z , Yunxiang T , Zhiping L , Zhimin Y . KNTC1 knockdown suppresses cell proliferation of colon cancer. 3. Biotech. 2021;11:262. 10.1007/s13205-021-02800-0. PMC8113418 33996374

[33] Gu C , Wang Y , Zhang L , Qiao L , Sun S , Shao M , et al. AHSA1 is a promising therapeutic target for cellular proliferation and proteasome inhibitor resistance in multiple myeloma. J Exp Clin Cancer Res. 2022;41:11. 10.1186/s13046-021-02220-1. 34991674 PMC8734095

[34] Shin DS , Zaretsky JM , Escuin‐Ordinas H , Garcia‐Diaz A , Hu‐Lieskovan S , Kalbasi A , et al. Primary Resistance to PD‐1 Blockade Mediated by JAK1/2 Mutations. Cancer Discov. 2017;7:188–201. 10.1158/2159-8290.CD-16-1223. 27903500 PMC5296316

[35] Zaretsky JM , Garcia‐Diaz A , Shin DS , Escuin‐Ordinas H , Hugo W , Hu‐Lieskovan S , et al. Mutations associated with acquired resistance to PD‐1 blockade in melanoma. N Engl J Med. 2016;375:819–29. 10.1056/NEJMoa1604958. 27433843 PMC5007206

[36] Ivashkiv LB , Donlin LT . Regulation of type I interferon responses. Nat Rev Immunol. 2014;14:36–49. 10.1038/nri3581. 24362405 PMC4084561

[37] Lee M , Lee Y , Song J , Lee J , Chang SY . Tissue‐specific Role of CX3CR1 expressing immune cells and their relationships with human disease. Immune Netw. 2018;18:e5. 10.4110/in.2018.18.e5. 29503738 PMC5833124

[38] Tamura R , Yoshihara K , Nakaoka H , Yachida N , Yamaguchi M , Suda K , et al. XCL1 expression correlates with CD8‐positive T cells infiltration and PD‐L1 expression in squamous cell carcinoma arising from mature cystic teratoma of the ovary. Oncogene. 2020;39:3541–54. 10.1038/s41388-020-1237-0. 32115573 PMC7176584

[39] Arora A , Olshen AB , Seshan VE , Shen R . Pan‐cancer identification of clinically relevant genomic subtypes using outcome‐weighted integrative clustering. Genome Med. 2020;12:110. 10.1186/s13073-020-00804-8. 33272320 PMC7716509

[40] Liu W , Zhuang C , Huang T , Yang S , Zhang M , Lin B , et al. Loss of CDKN2A at chromosome 9 has a poor clinical prognosis and promotes lung cancer progression. Mol Genet Genomic Med. 2020;8:e1521. 10.1002/mgg3.1521. 33155773 PMC7767555

[41] Roy DM , Walsh LA , Desrichard A , Huse JT , Wu W , Gao J , et al. Integrated genomics for pinpointing survival loci within arm‐level somatic copy number alterations. Cancer Cell. 2016;29:737–50. 10.1016/j.ccell.2016.03.025. 27165745 PMC4864611

[42] Davoli T , Uno H , Wooten EC , Elledge SJ . Tumor aneuploidy correlates with markers of immune evasion and with reduced response to immunotherapy. Science. 2017;355:25. 10.1126/science.aaf8399. 28104840 PMC5592794

[43] Shukla A , Nguyen THM , Moka SB , Ellis JJ , Grady JP , Oey H , et al. Chromosome arm aneuploidies shape tumour evolution and drug response. Nat Commun. 2020;11:449. 10.1038/s41467-020-14286-0. 31974379 PMC6978319

[44] Stark GR , Darnell JE . The JAK‐STAT pathway at twenty. Immunity. 2012;36:503–14. 10.1016/j.immuni.2012.03.013. 22520844 PMC3909993

[45] Bugter JM , Fenderico N , Maurice MM . Mutations and mechanisms of WNT pathway tumour suppressors in cancer. Nat Rev Cancer. 2021;21:5–21. 10.1038/s41568-020-00307-z. 33097916

[46] Mugarza E , van Maldegem F , Boumelha J , Moore C , Rana S , Sopena ML , et al. Therapeutic KRAS G12C inhibition drives effective interferon‐mediated anti‐tumour immunity in immunogenic lung cancers. bioRxiv. 2021;2010:464819. 10.1101/2021.10.18.464819. PMC9299537 35857848

[47] Muthalagu N , Monteverde T , Raffo‐Iraolagoitia X , Wiesheu R , Whyte D , Hedley A , et al. Repression of the Type I Interferon Pathway Underlies MYC‐ and KRAS‐Dependent Evasion of NK and B Cells in Pancreatic Ductal Adenocarcinoma. Cancer Discov. 2020;10:872–87. 10.1158/2159-8290.CD-19-0620. 32200350 PMC7611248

[48] Zimmerli D , Brambillasca CS , Talens F , Bhin J , Bhattacharya A , Joosten SEP , et al. MYC promotes immune‐suppression in TNBC via inhibition of IFN signaling. bioRxiv. 2021;2002:432659. 10.1101/2021.02.24.432659.

