# Supplementary figures and images for: Co‐occurrence of CDKN2A/B and IFN‐I homozygous deletions correlates with an immunosuppressive phenotype and poor prognosis in lung adenocarcinoma
Source: Mol Oncol. 2022 Mar 15;16(8):1746–60. doi: 10.1002/1878-0261.13206 (PMC9019898; doi:10.1002/1878-0261.13206)

A

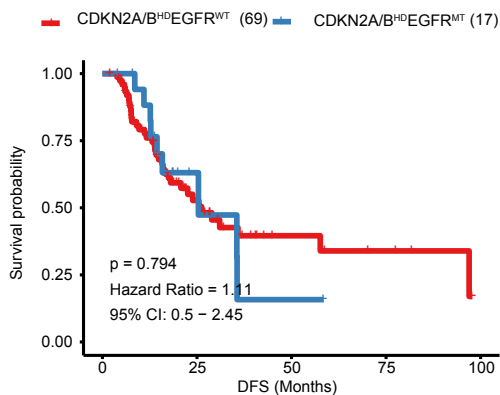

B

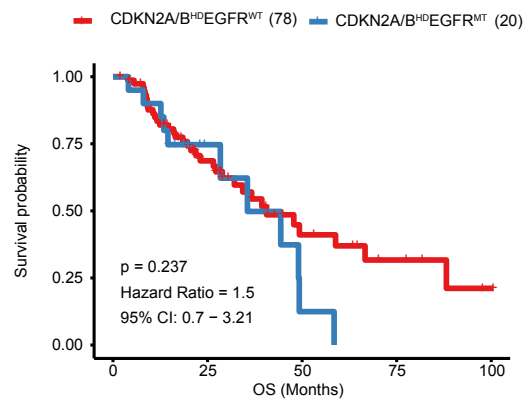

C

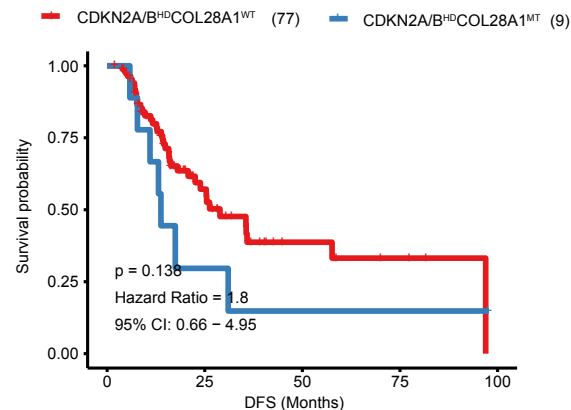

D

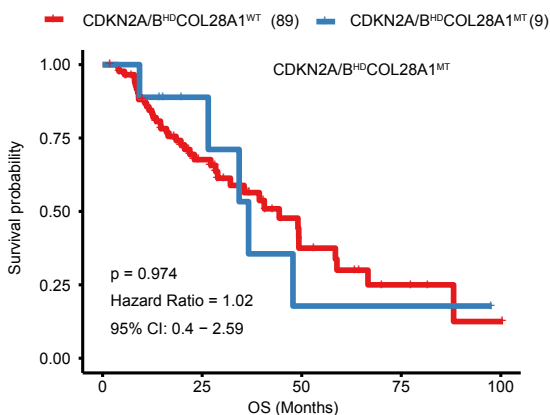

E

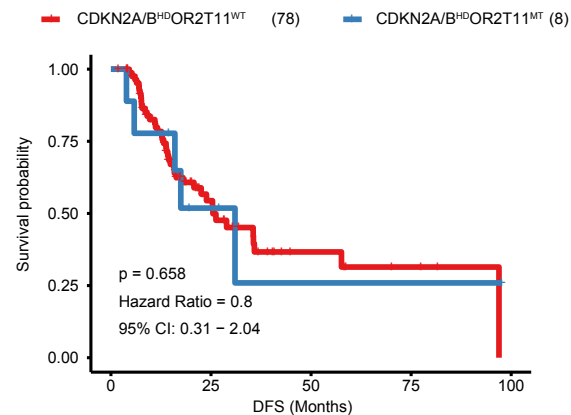

F

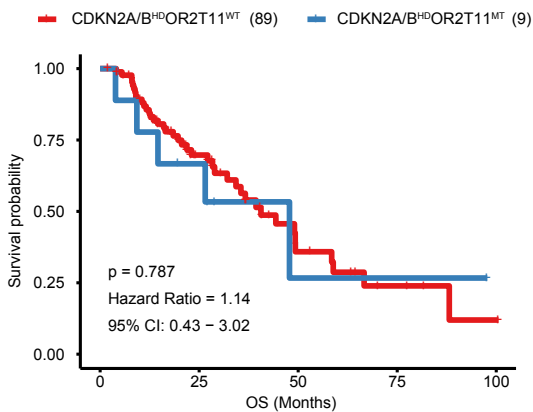

G

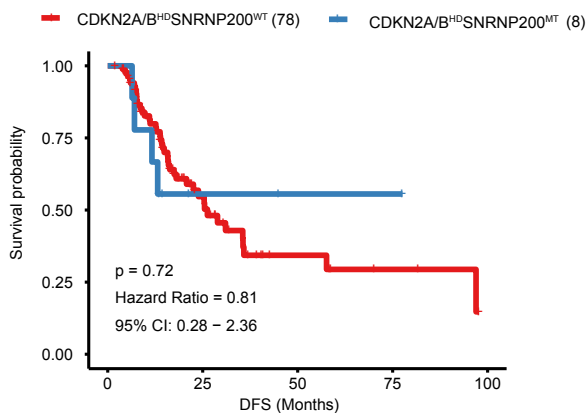

H

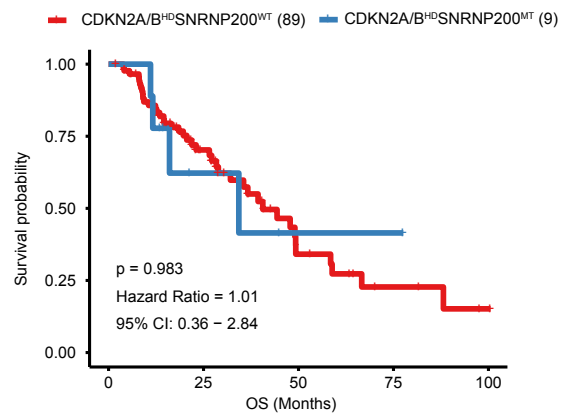

Supplement: Supplementary file 1 — Fig S1. Survival of patients with CDKN2A/B homozygous deletion and co‐occurring mutation in other genes. The log‐rank test was used to compare the survival times between two groups. [file MOL2-16-1746-s002.pdf]

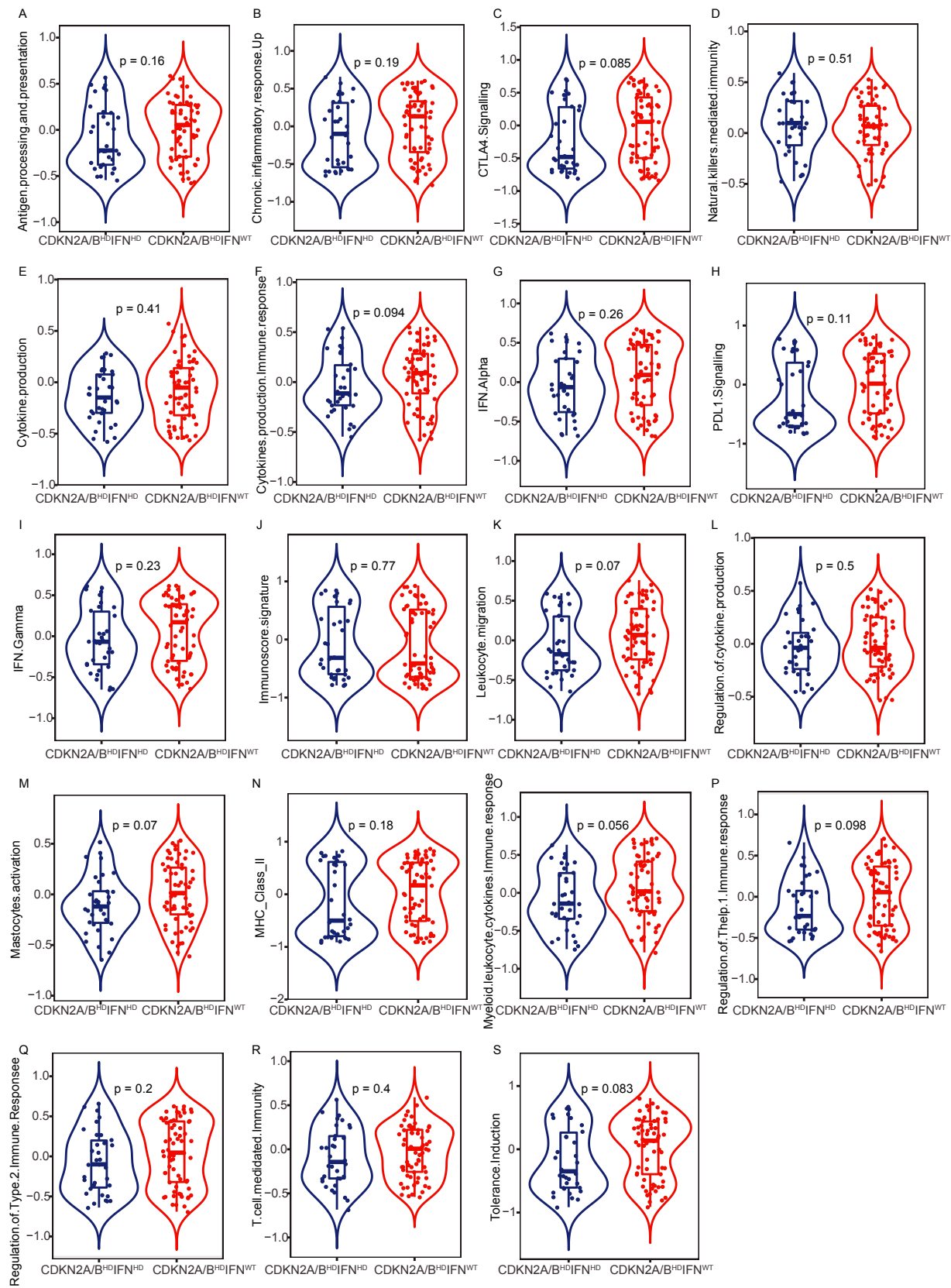

Supplement: Supplementary file 2 — Fig S2. Comparison of immune‐related gene sets with no significant difference in enrichment score between two different IFN‐I CNV statuses in patients with CDKN2A/B homozygous deletion. [file MOL2-16-1746-s001.pdf]
